# An Initial Indonesian Genome-Wide SNP-Array Study with Functional Variant Prioritization Reveals NASP and GPR78 Candidate SNVs in Hepatocellular Carcinoma

**DOI:** 10.3390/biomedicines14040931

**Published:** 2026-04-20

**Authors:** Toar Jean Maurice Lalisang, Vania Myralda Giamour Marbun, Linda Erlina, Nathaniel Jason Zacharia, Kezia Nathania Limbong Allo, Fadilah Fadilah, Aisyah Fitriannisa Prawiningrum

**Affiliations:** 1 Digestive Surgery Division, Faculty of Medicine, Cipto Mangunkusumo Hospital, Universitas Indonesia, Jakarta 10430, Indonesia; vania.myralda@gmail.com (V.M.G.M.); zachariajason8@gmail.com (N.J.Z.); kezianathania97@yahoo.co.id (K.N.L.A.); 2 Department of Medical Chemistry, Faculty of Medicine, Universitas Indonesia, Jakarta 10430, Indonesia; linda.erlina22@ui.ac.id (L.E.); fadilah.msi@ui.ac.id (F.F.); aisyahfitriannisa@gmail.com (A.F.P.); 3 Bioinformatics Core Facilities, Indonesian Medical Education and Medical Research Institute (IMERI), Faculty of Medicine, Universitas Indonesia, Jakarta 10430, Indonesia

**Keywords:** functional variant prioritization, HCC, SNPs, NASP, GPR78, Indonesia

## Abstract

**Background/Objectives**: Population-specific genomic data are essential for understanding hepatocellular carcinoma (HCC) biology, particularly in underrepresented regions. This study aimed to perform exploratory single-nucleotide polymorphism (SNP)-array-based profiling of HCC tumor samples from Indonesian patients and to prioritize candidate functional variants using a systematic in silico framework. **Methods**: This retrospective cross-sectional study included 15 resected HCC cases with available formalin-fixed paraffin-embedded (FFPE) tumor tissue. Genome-wide SNP genotyping was performed using the Illumina Asian Screening Array. Following quality control and filtering, variants were annotated using the Ensembl Variant Effect Predictor. A case-only functional prioritization approach incorporating multiple in silico prediction tools was applied, followed by gene-level burden aggregation. **Results**: After multistep filtering, 11 samples and 104 prioritized variants were retained for analysis. Variants consisted predominantly of splice-region, missense, and regulatory changes. Gene-level burden analysis identified Nuclear Autoantigenic Sperm Protein (NASP, rs775916096) as the highest-ranked candidate gene, while G protein-coupled receptor 78 (GPR78, rs558447540) emerged as a secondary candidate with predicted functional annotations but currently limited biological evidence in HCC. Given the tumor-only design without matched normal tissue, the prioritized variants cannot be distinguished from rare germline variants. **Conclusions**: This exploratory SNP-array study provides a hypothesis-generating framework for functional variant prioritization in Indonesian HCC. NASP and GPR78 represent preliminary candidates that require validation in larger cohorts with matched normal tissue and sequencing-based confirmation.

## 1. Introduction

Hepatocellular carcinoma (HCC) is a biologically heterogeneous malignancy and remains one of the leading causes of cancer-related death worldwide. Treatment outcomes are determined not only by intrinsic tumor biology but also by the extent of preserved hepatic function. In Indonesia, the burden of HCC remains particularly substantial. In 2022, an estimated 23,805 new cases and 23,383 deaths were reported, underscoring the exceptionally high fatality of this malignancy, with mortality nearly approaching incidence [1]. Advances in genomic technologies have substantially improved our current understanding of HCC biology by elucidating key molecular pathways involved in hepatocarcinogenesis, thereby facilitating the identification of candidate biomarkers, therapeutic targets, and potential mechanisms of treatment resistance [2,3,4].

In settings characterized by distinct geographic backgrounds, population structure, and risk factor distributions, locally generated genomic data are essential for hypothesis generation and for informing the design of appropriately powered downstream validation studies [3,5]. Although high-depth sequencing remains the preferred approach for comprehensive mutation discovery, alternative genomic platforms may still yield informative genome-wide data at a more feasible scale. In this regard, genome-wide SNP array genotyping is a practical strategy for capturing tumor-derived variant signals from archived tissue specimens, particularly when integrated with stringent quality control and systematic functional annotation.

In this study, we report genome-wide SNP-array profiles derived from formalin-fixed paraffin-embedded tumor specimens obtained from Indonesian patients who underwent surgical resection for HCC at Dr. Cipto Mangunkusumo Hospital. We further implemented a transparent functional prioritization framework that incorporates established in silico prediction tools to nominate candidate functional variants, followed by case-only gene-level aggregation to rank genes by cumulative predicted deleterious burden. This study is intended as a hypothesis-generating resource rather than a case–control association analysis, with the aim of generating a rational shortlist of candidate genes and variants for subsequent validation using matched normal tissue, sequencing-based confirmation, and correlation with clinicopathologic characteristics and clinical outcomes.

## 2. Materials and Methods

**Study Design**

In this retrospective cross-sectional study, we included patients with hepatocellular carcinoma (HCC) who underwent hepatectomy at Dr. Cipto Mangunkusumo Hospital between 2007 and 2023 and for whom archived formalin-fixed paraffin-embedded (FFPE) tumor tissue was available for genomic analysis. Eligibility criteria comprised histopathologic confirmation of HCC in the resection specimen and adequate FFPE material for DNA extraction and SNP array genotyping. Clinical and pathological variables, including suspected etiologic risk factors, Child–Pugh class, and Barcelona Clinic Liver Cancer (BCLC) stage, were systematically abstracted from the medical records. Tumor characteristics were ascertained from operative reports, pathology records, and imaging findings, including anatomical location, maximal tumor diameter, tumor multiplicity, and other relevant morphologic features. Data on recurrence and surveillance were obtained from institutional follow-up records and, where necessary, reconfirmed through structured contact with patients or family members during the data collection period. Clinical data extraction and genome-wide SNP-array genotyping were performed in November 2024. Prior to analysis, all datasets were de-identified, and only anonymized data were accessible to the investigators.

**FFPE DNA Extraction & Purification**

Genomic DNA was isolated from FFPE tumor tissue using the QIAamp DNA FFPE Tissue Kit (Qiagen, Hilden, Germany) according to the manufacturer’s protocol. For each sample, six 10 µm-thick sections were processed. Following xylene-based deparaffinization, tissue was lysed under denaturing conditions with proteinase K. To reverse formalin-induced cross-linking, lysates were incubated at 90 °C prior to column-based purification. DNA was subsequently captured on a silica membrane, washed to remove residual contaminants, and eluted in 20 µL of the supplied elution buffer. DNA concentration and purity were evaluated before downstream analysis using NanoDrop spectrophotometry and Qubit 4.0 fluorometry.

Because FFPE-derived DNA is frequently limited in quantity and may be contaminated with residual impurities, an additional cleanup and concentration step was performed using the DNA Clean & Concentrator-5 kit (Zymo Research, Irvine, CA, USA). DNA Binding Buffer was added at 2–7 times the sample volume, and the mixture was loaded onto a Zymo-Spin column for centrifugation-based DNA capture. After two washes with DNA Wash Buffer, DNA was eluted in 8 µL of DNA Elution Buffer or nuclease-free water following a brief incubation at room temperature. For SNP-array genotyping, DNA input was normalized to 50 ng per sample whenever possible; when DNA yield was insufficient, the maximum recoverable amount was used. Purified DNA was stored at −20 °C until genotyping.

**SNP Array Genotyping**

Genome-wide SNP genotyping was conducted using the Infinium Asian Screening Array (ASA) BeadChip (Illumina, San Diego, CA, USA) following the manufacturer’s standard Infinium assay protocol. In brief, genomic DNA was subjected to whole-genome amplification with an incubation at 37 °C for 20–24 h, followed by 1 h of enzymatic fragmentation. The amplified DNA was then precipitated at 4 °C for 30 min and resuspended before hybridization. Samples were hybridized to the BeadChip at 48 °C for 16–24 h. After post-hybridization washing, single-base extension and staining were carried out at 44 °C in accordance with the Infinium workflow. Arrays were scanned using the Illumina iScan platform to generate raw intensity data (IDAT files), which were subsequently used for genotype calling and downstream quality control.

**SNP-Array Preprocessing and Quality Control (GenomeStudio and PLINK)**

Genome-wide SNP-array data generated from FFPE tumor-derived DNA were processed using a standardized genotyping quality control workflow. Raw intensity (IDAT) files were imported into GenomeStudio v2.0 (Illumina) for genotype calling and initial sample-level assessment. Sample quality was evaluated using the call rate and the P10 GC metric, and the call rate versus P10 GC plot was visually inspected to identify outlier samples. Samples falling outside the main cluster or exhibiting a call rate below 0.80 were excluded. SNP statistics were subsequently recalculated in GenomeStudio, and low-quality markers flagged by the software were removed prior to data export.

GenomeStudio output files were then converted to PLINK binary format (BED/BIM/FAM) using PLINK v1.9.0-b.7.7 with the --make-bed option. Individual- and variant-level missingness were assessed using individual missingness (IMISS) and locus/SNP missingness (LMISS) metrics, and samples or SNPs with missingness greater than 0.20 were excluded (--mind 0.2, --geno 0.2). Analyses were restricted to autosomal SNPs (chromosomes 1–22). To reduce redundancy in downstream gene-level aggregation analyses, linkage disequilibrium pruning was performed using --indep-pairwise 50 5 0.2.

Given the tumor-only design and the absence of matched normal tissue, downstream analyses were structured to minimize reliance on population-genetic assumptions. Accordingly, the core quality control framework was based primarily on genotyping performance metrics, including call rate, missingness, and linkage disequilibrium pruning, whereas population allele frequency filtering was applied principally during the annotation stage using external reference databases. Hardy–Weinberg equilibrium and minor allele frequency filters were evaluated only as supplementary quality control heuristics to identify potential technical artifacts and were not interpreted as biologically expected features of tumor-derived genotypes. Following quality control, genotype data were converted to Variant Call Format (VCF) using GTC2VCF for downstream annotation.

Overall, the dataset comprised 655,366 SNPs before quality control, of which 104 remained after the application of the GenomeStudio- and PLINK-based filtering pipeline.

**Annotation and Functional Filtering**

Variant annotation was performed using the Ensembl Variant Effect Predictor (VEP; GRCh38, web interface) to assign each SNP to its corresponding gene context and to predict its functional consequence. In this study, specific variants are identified using their Reference SNP cluster ID (rs number), which is the standardized nomenclature assigned by the NCBI dbSNP database indicating the specific chromosomal position and allele changes. The potential deleterious impact of missense variants on protein function was assessed using Sorting Intolerant From Tolerant (SIFT), PolyPhen-2, Combined Annotation Dependent Depletion (CADD), and Rare Exome Variant Ensemble Learner (REVEL), whereas the putative effects of splice-region variants on pre-mRNA splicing were evaluated using SpliceAI and MaxEntScan [6,7,8,9,10]. Population allele frequency data were obtained from the Genome Aggregation Database (gnomAD), and variants were defined as rare when the global allele frequency was <0.01. No putative loss-of-function variants were identified in the analyzed dataset; therefore, functional prioritization was focused on predicted splice-disrupting variants, deleterious missense variants, and selected regulatory variants retained for exploratory hypothesis generation.

**Weighting Scheme**

Each variant was assigned a functional weight based on predicted molecular impact:

1. Variants with strong evidence of splice disruption, defined as a maximum SpliceAI delta score ≥ 0.5 or a MaxEntScan splice site score difference ≤ −3, were assigned a weight of 3.
2. Damaging missense variants were assigned a weight of 2 and were defined as missense variants with REVEL ≥ 0.5 or CADD PHRED ≥ 20, supported by a deleterious SIFT prediction or a damaging PolyPhen-2 prediction.
3. Regulatory or untranslated region variants, including upstream, promoter-annotated, or untranslated region (UTR) variants, were assigned a weight of 1.
4. Variants not meeting these criteria were assigned a weight of 0.

**Gene-Level Burden Analysis**

In the absence of individual-level genotype identifiers and an external control cohort, we applied a case-only gene-level burden ranking framework to capture the cumulative predicted functional impact of variants within each gene. For each gene, a raw burden score was calculated by summing the weights of all mapped variants. To stabilize the distribution and reduce the influence of extreme values, the raw burden score was log-transformed as follows:

Log Burden = ln (1 + raw burden score)

Genes were then ranked by their log-transformed burden scores, and candidate genes were defined as those in the upper 5th percentile of the ranked distribution for downstream interpretation and exploratory hypothesis generation.

## 3. Results

### 3.1. Patient Clinical Characteristics

A total of 15 patients with HCC who underwent hepatectomy were included in the study. The cohort was predominantly male and exhibited generally preserved hepatic function, with the majority of patients classified as Child–Pugh class A. Tumors involved multiple hepatic segments, most frequently segments II, III, and VIII. Hepatitis B virus infection was the most common underlying risk factor, followed by hepatitis C virus infection, nonalcoholic steatohepatitis, and no clearly documented etiology. Most patients presented with early- to intermediate-stage disease, corresponding to BCLC stage A or B. Tumor size most commonly ranged from 5 to 10 cm, and histopathologic evaluation most often showed moderate tumor differentiation (Table 1).

During follow-up, HCC recurrence was documented in three patients. Overall survival remained consistent throughout the first year after surgery, followed by stepwise declines at approximately 19, 22, 26, and 33 months, corresponding to subsequent deaths. Several patients were censored during follow-up, including long-term survivors with follow-up extending to 41 months. Observed overall survival ranged from 16 to 41 months, and median overall survival was not reached within the study period, indicating that more than half of the cohort remained alive at the last follow-up.

### 3.2. DNA Extraction and SNP-Array Genotyping

DNA extraction was successful in all 15 FFPE tumor samples. The A260/280 purity ratio ranged from 1.73 to 2.07, and the DNA concentration ranged from 0.678 to 37.7 ng/µL. All samples subsequently underwent SNP-array processing using the Infinium Asian Screening Array (ASA) BeadChip (Illumina), and the arrays were scanned using the Illumina iScan platform. Initial genotype calling in GenomeStudio v2.0 identified 655,366 SNP markers across 15 patients, comprising 12 male and 3 female individuals.

### 3.3. Overview of Workflow and Yield

**Figure 1:**
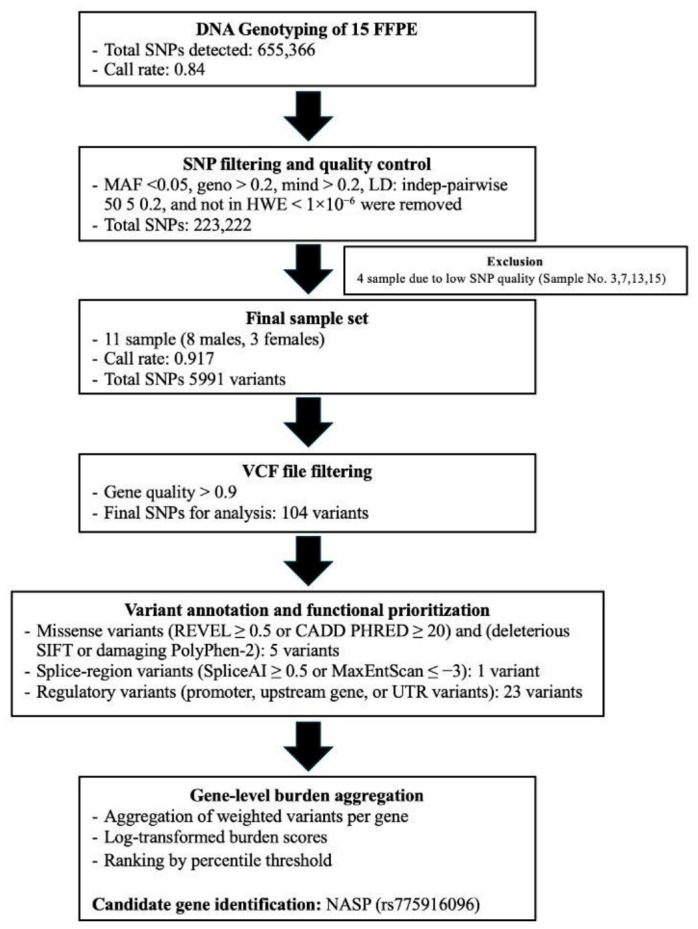
Workflow of SNP genotyping, quality control, and functional prioritization. Sequential filtering and annotation steps reduced the initial variant set to identify prioritized candidate genes based on functional impact and gene-level burden analysis. FFPE, formalin-fixed paraffin-embedded; SNP, single nucleotide polymorphism; MAF, minor allele frequency; LD, linkage disequilibrium; HWE, Hardy–Weinberg Equilibrium; VCF, Variant Call Format; REVEL, Rare Exome Variant Ensemble Learner; CADD, Combined Annotation Dependent Depletion; SIFT, Sorting Intolerant From Tolerant; UTR, untranslated region; NASP, Nuclear Autoantigenic Sperm Protein.

Genome-wide SNP-array profiling was performed on 15 FFPE tumor specimens from patients with HCC, yielding 104 prioritized variants after sequential quality control, annotation, and functional filtering. From an initial total of 655,366 SNP markers, a multistep analytical pipeline progressively reduced the dataset to a final subset of variants retained for downstream functional prioritization and case-only gene-level aggregation. A schematic summary of the analytical workflow is provided in Figure 1.

In brief, SNP-array genotyping generated 655,366 markers across 15 samples, corresponding to an overall genotyping rate of 0.84365. After GenomeStudio- and PLINK-based preprocessing and marker-level refinement, 223,222 SNPs were retained. Four low-quality samples (sample IDs 3, 7, 13, and 15) were excluded because of poor technical quality, resulting in a final analytic dataset of 11 samples with an overall call rate of 0.917923. Subsequent VCF conversion and additional genotype-level quality filtering further reduced the dataset to 5991 SNPs, of which 104 variants remained after downstream annotation and functional filtering.

### 3.4. SNP-Array Quality Control and Sample/Marker Filtering

**Figure 2:**
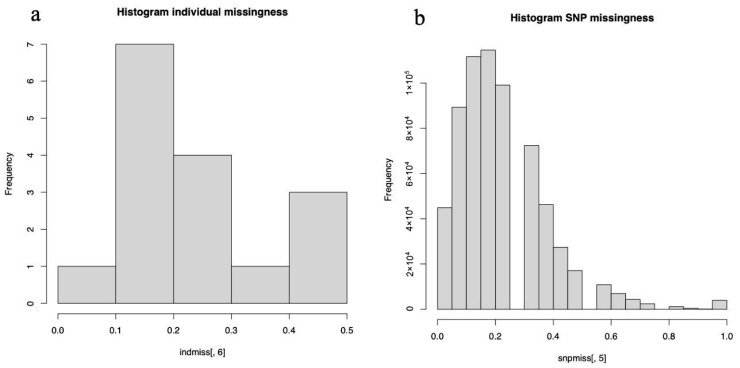
Distribution of missing genotype data across samples and variants. (**a**) Histogram showing the proportion of missing genotypes per individual, reflecting sample-level data completeness. (**b**) Histogram showing the proportion of missing genotypes per single-nucleotide polymorphism (SNP), illustrating variant-level missingness across the dataset. These distributions were used to assess data quality prior to downstream genetic analyses and to inform the selection of appropriate quality control thresholds.

**Figure 3:**
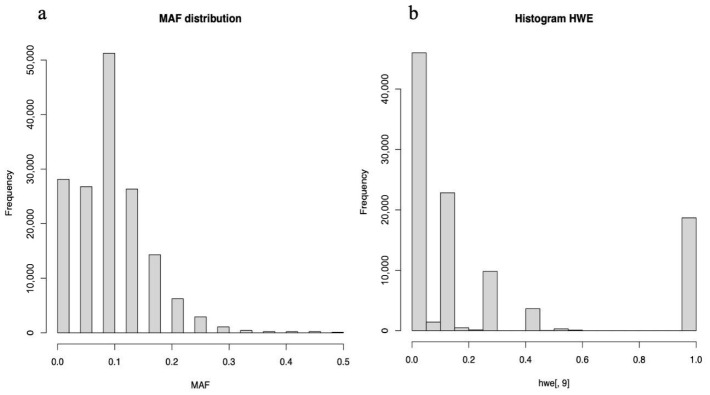
Distribution of variant-level allele frequency and Hardy–Weinberg equilibrium statistics. (**a**) Histogram of minor allele frequency (MAF) across all single-nucleotide polymorphisms (SNPs), illustrating the allele frequency spectrum of the dataset. (**b**) Histogram of Hardy–Weinberg equilibrium (HWE) test *p*-values for all SNPs, used to assess genotype quality and identify variants potentially affected by genotyping error, population stratification, or selection prior to quality control filtering.

Marker- and sample-level quality control was performed using GenomeStudio and PLINK, with the principal filtering steps summarized in Figure 2 and Figure 3. Initial quality assessment focused on genotyping performance metrics, including call rate, P10 GC, and sample- and variant-level missingness. Samples falling outside the main call rate versus P10 GC cluster or showing a call rate below 0.80 were excluded in GenomeStudio. In PLINK, samples and SNPs with missingness greater than 0.20 were excluded using the --mind 0.2 and --geno 0.2 thresholds, and analyses were restricted to autosomal SNPs. Linkage disequilibrium pruning (--indep-pairwise 50 5 0.2) was then applied to reduce redundancy in downstream gene-level aggregation analyses.

Because this study used a tumor-only design without matched normal tissue, population-genetic filters, such as minor allele frequency and Hardy–Weinberg equilibrium, were treated as supplementary technical heuristics and were not interpreted as biologically expected properties of the dataset. After these preprocessing and refinement steps, 223,222 SNPs were retained. Four samples (all male; sample IDs 3, 7, 13, and 15) were excluded because of low genotyping quality. The remaining 11 samples (8 male and 3 female) showed an improved overall call rate of 0.917923, and 5991 SNPs were retained for downstream analysis after final quality filtering.

### 3.5. Variant-Level Filtering and Functional Prioritization

Following the conversion of genotype data to Variant Call Format (VCF), variants were filtered using a genotype quality threshold of GQ > 0.9, resulting in 104 variants eligible for downstream annotation and functional prioritization. After annotation and functional filtering, most variants were excluded because they were not predicted to have meaningful functional consequences. No high-confidence loss-of-function variants were detected. The retained variants were predominantly splice-region, missense, and regulatory or untranslated-region variants (Table 2), which were subsequently prioritized using the prespecified weighting framework for downstream gene-level aggregation.

### 3.6. Gene-Level Burden Ranking and Key Candidates

Gene-level aggregation of weighted variants demonstrated a sparse distribution of predicted functional burden across the analyzed genes. Nuclear Autoantigenic Sperm Protein (NASP) was the highest-ranked gene and surpassed the prespecified upper 5th percentile threshold, predominantly driven by rs775916096, which satisfied the criteria for predicted functional significance within the splice/missense/regulatory prioritization framework. G protein-coupled receptor 78 (GPR78) (rs558447540) emerged as a secondary candidate gene, with predicted functional annotations in the missense and regulatory categories, although its cumulative burden score was lower than that observed for NASP. Taken together, the case-only gene-level burden analysis prioritized NASP (rs775916096) as the principal candidate gene and GPR78 (rs558447540) as a secondary candidate for downstream validation and biological interpretation.

Overall, the case-only gene-level burden analysis prioritized NASP (rs775916096) as the leading candidate gene, demonstrating the highest cumulative predicted functional burden across the analyzed dataset. This signal was primarily driven by variants classified as splice-related, missense, and regulatory. GPR78 (rs558447540) was identified as a secondary candidate gene, with predicted functional variants in the missense and regulatory categories, although its aggregate burden was lower than that observed for NASP.

## 4. Discussion

In this exploratory study, we performed genome-wide SNP-array profiling of FFPE-derived tumor DNA from an Indonesian HCC cohort and applied a structured functional prioritization framework to identify candidate variants [8,9]. From an initial set of 655,366 SNP markers, a multistep pipeline integrating genotyping quality control, variant-level filtering, and in silico annotation yielded 104 prioritized variants across 11 analyzable cases. Gene-level aggregation revealed a sparse distribution of predicted functional burden, highlighting a small number of candidate genes for downstream interpretation.

Among these, NASP emerged as the highest-ranked candidate gene, driven primarily by rs775916096. NASP encodes a histone-binding protein involved in chromatin assembly, cell cycle progression, and DNA replication. Dysregulation of chromatin organization is a well-recognized hallmark of cancer, and accumulating evidence suggests that aberrant histone chaperone activity may contribute to oncogenic transcriptional programs and genomic instability. Although NASP has not been extensively characterized in hepatocellular carcinoma, prior studies in other malignancies and experimental models suggest a potential role in promoting cellular proliferation and tumor growth. In this context, the prioritization of NASP in our dataset provides a biologically plausible signal that warrants further functional validation [11,12,13,14].

Importantly, the functional annotation of rs775916096 reflects transcript-dependent consequences across multiple isoforms, including splice-region and coding/regulatory contexts. Such multi-layered annotation is expected in genome-wide analyses and does not imply simultaneous effects within a single transcript. Rather, it suggests that the variant may exert context-specific functional effects, with splice-region disruption representing a plausible primary mechanism that should be prioritized in downstream experimental studies.

A secondary candidate signal was identified in GPR78 (rs558447540), supported by predicted missense and regulatory annotations. GPR78 is an orphan G protein-coupled receptor with limited functional characterization, and its role in hepatocellular carcinoma remains largely unexplored. Unlike canonical HCC-associated genes, there is currently no substantial evidence linking GPR78 to hepatocarcinogenesis, tumor progression, or therapeutic response [15]. Therefore, this finding should be interpreted cautiously as a novel, hypothesis-generating signal, rather than as a biologically established contributor to HCC. Future studies integrating transcriptomic, proteomic, and functional assays will be necessary to determine whether GPR78 plays a meaningful role in liver tumor biology.

Notably, well-established HCC driver genes commonly reported in Asian cohorts, such as Tumor Protein 53 (TP53), Catenin Beta 1 (CTNNB1), Telomerase Reverse Transcriptase (TERT), Axis Inhibition Protein 1 (AXIN1), and AT-rich interacting domain-containing protein 1A (ARID1A) were not identified in the prioritized dataset [16]. This observation likely reflects the inherent limitations of SNP-array platforms, which are not optimized for comprehensive mutation discovery and may not capture key somatic alterations outside of predefined probe regions. In addition, the relatively small sample size further reduces the likelihood of detecting recurrent driver events. These factors underscore that the present study is not designed to identify canonical drivers, but rather to generate a prioritized shortlist of candidate variants for subsequent investigation.

From a methodological perspective, the use of SNP-array genotyping in FFPE-derived tumor samples represents a pragmatic approach in settings where sequencing resources may be limited and DNA quality is suboptimal. While next-generation sequencing provides higher resolution, SNP-array platforms can still yield informative genome-wide signals when combined with rigorous quality control and systematic functional annotation [17]. In this study, the integration of multiple prediction tools (SIFT, PolyPhen-2, CADD, REVEL, SpliceAI, and MaxEntScan) within a transparent weighting framework allowed for structured prioritization of variants based on predicted functional impact [6,7,8,9,10]. However, it should be emphasized that such in silico predictions are probabilistic and require experimental validation.

The clinicopathological profile of patients in this series generally reflected typical hepatocellular carcinoma characteristics, including a predominance of hepatitis B infection, preserved liver function, and intermediate to large tumor size at presentation. Nevertheless, the small sample size limits the interpretability of these findings, and the results should be considered descriptive only without statistical or population-level significance.

Several important limitations should be acknowledged. First, the tumor-only design without matched normal tissue precludes the distinction between somatic mutations and rare germline variants. As a result, the prioritized variants may represent population-specific rare variants rather than tumor-specific alterations. Second, the small sample size (n = 11 after quality control) limits the robustness and generalizability of the findings and increases the risk of stochastic signals. Third, SNP-array platforms provide limited genomic coverage compared with sequencing-based approaches and may fail to detect key driver mutations. Fourth, no orthogonal validation using sequencing was performed, and therefore the accuracy of variant calls cannot be independently confirmed. Finally, clinical correlation analyses in this cohort remain descriptive and should not be interpreted as evidence of prognostic association.

Taken together, this study should be viewed as a pilot, hypothesis-generating analysis that demonstrates the feasibility of SNP-array-based functional variant prioritization in an Indonesian HCC cohort. The identification of NASP as a top-ranked candidate and GPR78 as a secondary exploratory signal provides a focused starting point for future studies. Subsequent work should incorporate larger, multicenter cohorts, matched normal tissue for somatic variant calling, sequencing-based validation, and integration with transcriptomic and protein-level data to establish biological relevance.

## 5. Conclusions

This study demonstrates the feasibility of applying a SNP-array–based functional variant prioritization framework to FFPE-derived tumor samples from an Indonesian hepatocellular carcinoma cohort. Within this exploratory, tumor-only design, NASP (rs775916096) emerged as the top-ranked candidate gene, while GPR78 (rs558447540) was identified as a secondary, hypothesis-generating signal with currently limited biological evidence in HCC.

Given the absence of matched normal tissue, the small sample size, and the lack of sequencing-based validation, these findings should be interpreted with caution and should not be considered evidence of disease-associated driver events. Rather, this study provides a preliminary, population-relevant shortlist of candidate variants for further investigation.

Future studies incorporating larger cohorts, matched tumor-normal analysis, and multi-omic validation will be essential to determine the biological and clinical relevance of these prioritized variants. Overall, this work represents an initial step toward generating population-specific genomic insights into Indonesian HCC and provides a foundation for future precision oncology research in this setting.

## Figures and Tables

**Table 1:** Clinicopathological Characteristics, Demographic Features (Age and Sex), and Selected SNPs in Patients with Hepatocellular Carcinoma.

Case	Age (Sex)	Location (Segment)	Risk Factor	Child Pugh	BCLC	Diameter	Differentiation	Recurrency	Survival (Months)
1	61 (M)	II	Hep B	A	A	5–10 cm	Moderate	No	32
2	53 (M)	II, III	No risk factor	A	C	>10 cm	Well	No	41
3 *	46 (M)	VIII	Hep B	A	B	5–10 cm	Moderate	No	26
4	38 (M)	II, III, IV	No risk factor	A	C	>10 cm	Moderate	No	26 (Died)
5	79 (M)	II, III	No risk factor	A	B	5–10 cm	Moderate	No	19 (Died)
6	74 (F)	III	Hep C	A	B	5–10 cm	Well	No	22 (Died)
7 *	63 (M)	V, VIII	Hep B	A	B	5–10 cm	Moderate	No	9
8	66 (M)	II, III, IV	Hep B	A	A	>10 cm	Moderate	No	2 Days (OM)
9	48 (M)	III	NASH	A	B	<5 cm	Moderate	No	16
10	40 (F)	V, VI, VII	Hep B	A	B	5–10 cm	Well	Yes	41
11	63 (M)	IVa, VIII	Hep C	A	B	>10 cm	Moderate	Yes	9
12	70 (F)	VII, VIII	Hep B & C	A	0	5–10 cm	Moderate	No	33
13 *	74 (M)	II, III	Hep B	A	A	>10 cm	Moderate	No	33 (Died)
14	45 (M)	V	Hep B	A	A	5–10 cm	Moderate	No	Lost to follow up
15 *	59 (M)	VII	Hep B	A	A	<5 cm	Moderate	Yes	24

SNP, single-nucleotide polymorphism; M, male; F, female; Hep B, hepatitis B virus infection; Hep C, hepatitis C virus infection; NASH, non-alcoholic steatohepatitis; BCLC, Barcelona Clinic Liver Cancer staging system; OM, operative mortality. * excluded cases.

**Table 2:** Functional Annotation of Candidate Genes Based on Missense, Splicing, and Regulatory Variants.

Gene	Missense	Splicing	Regulatory	SNPs
NASP	✔	✔	✔	rs775916096
GPR78	✔	–	✔	rs558447540.1; rs558447540
MTCL1	✔	–	–	rs753910817.1; rs753910817
ARHGEF37	✔	–	–	exm493941; rs116647383
ZBTB34	✔	–	–	rs764972186
LINC01755	–	–	✔	GSA-rs11206701; rs11206701
LINC02796	–	–	✔	1:72996462; rs144884896
UCK2	–	–	✔	rs4656467
TIGAR	–	–	✔	kgp538733; rs11063079
KCNA5	–	–	✔	rs11063479
SIAH1	–	–	✔	kgp2204559; rs111963469
MMP2-AS1	–	–	✔	kgp6450333; rs2540725
Y_RNA	–	–	✔	rs753910817.1; rs753910817
MIR4266	–	–	✔	2:109930939; rs13424751
SLC19A4P	–	–	✔	kgp6218746; rs73082830
PCYT1A	–	–	✔	kgp18150757; rs76185976
LINC01331	–	–	✔	kgp5073441; rs550872
PCDHGA6	–	–	✔	rs762773164.2; rs762773164
RNU6-588P	–	–	✔	exm493941; rs116647383
TGILR	–	–	✔	rs4074617
HLA-DQA2	–	–	✔	GSA-rs28421301; rs28421301
HLA-DMA	–	–	✔	rs1431394
MKRN6P	–	–	✔	rs2894401
MCPH1	–	–	✔	kgp531448; rs2515488
LINC02851	–	–	✔	9:6714335; rs189960320
EXOSC2	–	–	✔	9:133570021; rs144008421

SNP, single-nucleotide polymorphism. Missense variants refer to nonsynonymous coding changes; splicing variants include predicted splice donor/acceptor or splice-region alterations; regulatory variants include variants annotated in regulatory regions such as promoters, enhancers, or untranslated regions based on functional annotation databases.

## Data Availability

The data presented in this study are available on request from the corresponding author.

## Abbreviations

The following abbreviations are used in this manuscript:

ARID1A	AT-rich interacting domain−containing protein 1A
ASA	Asian Screening Array
AXIN1	Axis Inhibition Protein 1
BCLC	Barcelona Clinic Liver Cancer
CADD	Combined Annotation Dependent Depletion
CTNNB1	Catenin Beta 1
FFPE	Formalin-Fixed Paraffin-Embedded
gnomAD	Genome Aggregation Database
GPR78	G protein-coupled receptor 78
GQ	Genotype-Quality
HCC	Hepatocellular Carcinoma
HWE	Hardy–Weinberg Equilibrium
IMISS	Individual Missingness
LD	Linkage Disequilibrium
LMISS	Locus/SNP Missingness
MAF	Minor Allele Frequency
NASP	Nuclear Autoantigenic Sperm Protein
NF-κB	Nuclear Factor Kappa-Light-Chain-Enhancer of Activated B Cells
P10 GC	10% GenCall
REVEL	Rare Exome Variant Ensemble Learner
SIFT	Sorting Intolerant From Tolerant
SNP	Single Nucleotide Polymorphism
SNV	Single Nucleotide Variant
TERT	Telomerase Reverse Transcriptase
TP53	Tumor Protein 53
UTR	Untranslated Region
VEP	Variant Effect Predictor
VCF	Variant Call Format
